# Plastibell Device Circumcision versus Bone Cutter Technique in terms of Operative Outcomes and Parent’s Satisfaction

**DOI:** 10.12669/pjms.322.9510

**Published:** 2016

**Authors:** Tahir Mehmood, Hammad Azam, Muhammad Tariq, Zafar Iqbal, Hassan Mehmood, Syed Asif Hussain Shah

**Affiliations:** 1 Tahir Mehmood, FCPS General Surgery. Senior Registrar Pediatric Surgery, Sheikh Zayed Medical College and Hospital, Rahim Yaar Khan, Pakistan; 2 Hammad Azam, FCPS General Surgery. Senior Registrar Cardiac Surgery, Sheikh Zayed Medical College and Hospital, Rahim Yaar Khan, Pakistan; 3 Muhammad Tariq, FCPS General Surgery. Senior Registrar Surgery, Bahawal Victoria Hospital, Bahawalpur, Pakistan; 4 Zafar Iqbal, FCPS Peads. Surgery. Associate Prof. of Pediatric Surgery, Sheikh Zayed Medical College and Hospital, Rahim Yaar Khan, Pakistan; 5 Hassan Mehmood, FCPS General Surgery. Professor of Surgery, Sheikh Zayed Medical College and Hospital, Rahim Yaar Khan, Pakistan; 6 Syed AsifHussain Shah, MBBS. Resident General Surgery, Sheikh Zayed Medical College and Hospital, Rahim Yaar Khan, Pakistan

**Keywords:** Circumcision, Plastibell device, bone cutter method

## Abstract

**Objective:**

To compare the rate of complications of Plastibell and bone cutter circumcision technique and recognition of top worries and satisfaction rate in the mind of parents before and after the procedure of Plastibell device (PD) circumcision in infants less than 6 months of age.

**Methods:**

It was a descriptive prospective study conducted at department of surgery Sheikh Zayed Hospital, Rahim Yar Khan. Two hundred parents of infants of less than six months of age were recruited for this study. Infants were divided into two equal groups. Group I included Plastibell circumcision technique and Group II included Bone Cutter Circumcision technique. Data was analyzed using SPSS Version 17. Independent sample t-test and chi-square test was used to compare quantitative and qualitative variables respectively. P-value <0.05 was taken as significant difference.

**Results:**

Total number of two hundred infants were included in this study. Most common worries of parents about Plastibell Device circumcision were; fear of fever (42.0%). Fear of pain and bleeding (66.0%). Plastibell Device method was associated with less operation time and bleeding as compared to bone cutter method (P-value <0.0001 and <0.0001 respectively). Incidence of complications other than bleeding and infection was 3.0% in bone cutter method and 1.0% in Plastibell device method. Pain score was significantly less in plastibell device group (p-value <0.0001). Post-operatively, 98% parents showed complete procedural satisfaction in Plastibell group versus 87% parents in bone cutter one month after surgery (P-value 0.003). About 4% parents in bone cutter method group showed cosmetic displeasure versus only 1% parents in plastibell device group.

**Conclusion:**

The study concluded that Plastibell Device circumcision is a safer technique for circumcision and is associated with highest level of parent’s satisfaction.

## INTRODUCTION

Circumcision is a religious practice as well asasurgical procedure having definite risk and benefits.^1^ Circumcision is mostly performed for religious purposes not for medical reasons. In Pakistan, males undergo circumcision from 1^st^ day to five years of life. Circumcision is easily performed in both infants and young children.^2^ Healing is most commonly completed within 1^st^ two weeks after operation. It carries a low risk of surgical complications ranging from 1 to 15%.^3^,^4^

Out of various techniques available for circumcision, Plastibell device methodhas now become the method of choice in childrenless thanone year of age in the modern world. ThePlastibell circumcision device was introduced by Hollister in 1950s. It is a plastic ring having a handle designed for male circumcision and is available in ring sizes 1.1 to 1.7 cm. The rate of complications with the use of Plastibell device (PD) are reported to be 2.0-3.0% in some studies.^5^,^6^ Plastibell circumcision is largely under vision, almost blood less, least painful and easy to perform, with no tragic complications like traumatic amputation of glans, urethro-cutaneous fistula etc,^7^,^8^ which are frequently seen in bone cuter method.

According to a study, Plastibell device is mainly used by Pediatric surgeons in Pakistan but not by others who are performing circumcision on regular basis.^9^ Bone cutter method is a routinely used method for circumcision by general practitioners.^9^ According to a study, bone cutter method is still a most commonly used method for circumcision in Pakistan.^10^ There are very few randomized trials available in Pakistan regarding efficacy and safety of plastibell circumcision device over the bone cutter method, so general practitioners are reluctant to adopt this procedure for circumcision.

We conducted this study to compare the rate of complications of Plastibell and bone cutter circumcision technique and recognition of top worries and satisfaction rate in the mind of parents before and after the procedure of Plastibell device (PD) circumcision in infants less than 6 months of age.

## METHODS

It was a descriptive prospective study conducted at department of surgery Sheikh Zayed Hospital, Rahim Yar Khan after ethical approval from the Research and Development Support unit of the hospital. Two hundred parents of infants and infants of less than six months of age were included in this study. Babies of more than six months of age, or with urological anomalies, bleeding disorders, sickle cell anemia & hemolytic diseases were excluded from study.

An informed consent was taken from the parents of infants and they were informed about the Plastibell circumcision method. Then they were asked to fill a Performa about the worries of the parents regarding bone cutter and plastibell device circumcision. No Performa was used for bone cutter method because this is an older technique and most of the parents were familiar with the bone cutter method and were not easily willing to let their child go for plastibell circumcision. So they were asked to fill this perfoma so that their main worries can be recognized. The data of complications were not included in the performa because they were not aware of complications of bone cutter and plastibell circumcision method.

Both procedures were performed under local anesthesia using 2% lidocaine solution at a dose of 4mg/kg (administrated using a 27-guage needle). In the plastibell circumcision technique, the plastic bell which fits over 2/3 of the glanz was placed under the foreskin and over the glans surface. The device was secured with a cotton thread supplied with the plastibell device. The parents were advised to inform if the bell does not separates within 10 days after the procedure. In bone cutter technique, the bone cutter was applied to the prepuce and was kept clamped for one minute. Light pressure was applied on the perpendicular axis of the cut edges until the glanz fully released. The circumcision site was dressed circumferentially with sterile gauze, the edges of the gauze were fixed by adhesive plaster. The parents were asked for routine check up on 2^nd^ post-operative day and one month after the procedure.

Operating time and bleeding amount during the procedure was recorded. Post-operative complications were recorded on a performa. Bleeding amount was calculated by weighting the gauze piece before using and then after the procedure. The average weight of 1 ml of blood is about one gram. Visual analogue scale (VAS) was used to measure pain. The performa of post-operative pain was filled by parents on 2^nd^ post-operative day. Oral analgesia medication and a local analgesia ointment was given to all parents for management of pain and infection prevention to all parents. Then after one month of the procedure, the parents of both groups were asked to fill a Performa regarding overall satisfaction of the procedure including cosmetic concerns of the penis of baby.

Data was entered and analyzed using SPSS Version 17. Percentages were used to express qualitative variables. Independent sample t-test and chi-square test was used to compare quantitative and qualitative variables respectively. P-value <0.05 was taken as significant difference.

## RESULTS

Parents main worries regarding Plastibell Circumcision are shown in Table-I. Parent’s main worry about this technique was will there be a fever after circumcision? This fear was present in 42.0% parents. Other fear included pain and bleeding during the circumcision process and fear of night awakening with the child because of risk of bleeding or any other issue. The fear that their children will have any urinary problem after circumcision was only in 10.0% parents. Fear of cosmetic concerns i.e. regarding shape of penis using Plastibell device technique for surgery was found in 11.0% parents.

**Table-I:** Parent’s main worries before surgery regarding Plastibell Circumcision.

Parents main concerns	Number (%)
Fear of fever	42 (42.0%)
Fear of pain and bleeding	66 (66.0%)
fear of night awakening	8 (8.0%)
cosmetic concerns	11 (11.0%)
fear of urinary problems	10 (10.0%)

There was no significant difference in the age of babies at the time of surgery. The operation time was high in bone cutter method 5.25±1.48 minutes versus 3.03±0.87 minutes in Plastibell method (P-value <0.0001). There was more bleeding 10.65±3.31 ml in bone cutter method as compared to Plastibell method, in which bleeding was only 5.48±0.84 ml during operation (P-Value <0.0001). There was no incidence of infection in two groups. But there were three other complications in bone cutter method. These were; traumatic amputation of glans occurred in one baby, urethra-cutaneous fistula occurred in one baby and there was one incidence of over-circumcision. In Plastibell technique there was only one complication that was urine retention which required repositioning of the device. There was no incidence of delayed separation of plastibell device and bell impaction. Mean pain score was 3.14+1.19 in plastibell device group and 5.18+1.22 in bone cutter method and this difference was statistically highly significant (p-value <0.0001). Only 4% parents showed cosmetic displeasure about the shape of the penis in bone cutter method and 1% parents were dissatisfied in plastibell group. 98% parents showed complete satisfaction regarding the procedure in Plastibell group whereas only 87% parents in bone cutter method showed complete satisfaction one month after surgery, and this difference was statistically significant with P-value-0.003. So we found that Plastibell technique is associated with less operation time, bleeding and other complications and it is associated with higher parent’s satisfaction rate. Table-II

**Table-II:** Comparison of operative and post-operative variables and parents satisfaction rate.

Name of Variable	Plastibell Method	Bone Cutter Method	P-Value
Age (months) (mean±SD)	3.47+1.28	3.46+1.33	0.97
Operation time (mints) (mean±SD)	3.03±0.87	5.25±1.48	<0.0001
Bleeding (ml) (mean±SD)	5.48±0.84	10.65±3.31	<0.0001
Infection (%)	0.00	0.00	------
Pain score (mean±SD)	3.14±1.19	5.28±1.22	<0.0001
Other complications	1 (1.0%)	3 (3.0%)	0.31
Cosmetic displeasure (%)	1 (1.0%)	4 (4.0%)	0.17
Post-procedural Parentral Satisfaction (%)	98 (98%)	87 (87%)	0.003

Other Complications= amputation of glans, urethro-cutaneous fistula, Under circumcision, over circumcision, delayed separation of Plasti bell Device (PD), Bell Impection and urine retention due to PD.

## DISCUSSION

The word “circumcision” comes from Latin circum (meaning “around”) and cædere (meaning “to cut”). Early depictions of circumcision are found in paintings of Ancient Egyptian tombs.^11^,^12^ Religious male circumcision is considered a commandment from God in Judaism.^13^ According to the World Health Organization (WHO), global estimates suggest that 30% of males are circumcised, of whom 68% are Muslim.^14^

Circumcision using bone cutter method is blind with frequent tragic outcomes like amputation of glans and urethra-cutaneous fistula that leads to severe post-operative pain and in some cases intractable bleeding requiring with 3-0 absorbable sutures. Over circumcision and under circumcision are also common problems with bone cutter method.^7^ According to various studies, the rate of complications using bone cutter method varies from 4.7 to 8.4%.^15^,^16^ Whereas the rate of complications using plastibell device have been reported to be 2-3% only.^5^,^6^ Bleeding and local infection have been reported to be the most common complications of plastibell circumcision. Other complications that can occur are; bell impaction, incomplete separation of plastibell device, dysuria, and excessive loss of skin or inadequate skin removal.^17^

In this study, we found greater complication rate in bone cutter method. The bleeding rate was higher in bone cutter method as compared to plastibell device method in this study. Incidence of complications using bone cutter method in this study was 3.0% and only 1% in PD group. Traumatic amputation of the glans, urethrocutaneous fistula and over-circumcision were the main complications that occurred in bone cutter method. The only complication that occurred in PD group was urine retention. Freeman et al., showed 95.6% rate of parent satisfaction regarding plastibell circumcision.^18^ In our study the parent satisfaction rate was high; it was 98% in plastibell group and only 87% in bone cutter method group. Cosmetic displeasure was found only 1% parents in plastibell group and 4% parents in bone cutter method group.

Plastibell Device (PD) is associated with shorter operation time, less pain and smaller number of complications as compared to bone cutter method according to the results of this study. It is also associated with the highest level of parent’s satisfaction according to the results of our study. So this technique of circumcision should be used routinely as it is associated with least level of complications. As such the surgeon and the parents should not worry to adopt this technique.

## CONCLUSION

Plastibell Device circumcision is a safer technique and is associated with highest level of parent’s satisfaction.
